# The histone H3 lysine-27 demethylase UTX plays a critical role in colorectal cancer cell proliferation

**DOI:** 10.1186/s12935-019-0841-y

**Published:** 2019-05-22

**Authors:** Xin Tang, Wenwei Cai, Jing Cheng, Ping Lu, Shaojun Ma, Chaoting Chen, Yi Chen, Yun Sun, Caofeng Wang, Ping Hu, Xiaomin Lv, G. Sun, Yu Wang, Jing Sheng

**Affiliations:** 1 Departments of Geriatrics, Affiliated Ninth People’s Hospital, Shanghai Jiaotong University School of Medicine, Shanghai, China; 2 Departments of Gastroenterology, Affiliated Ninth People’s Hospital, Shanghai Jiaotong University School of Medicine, Shanghai, China

**Keywords:** UTX, CRC, Proliferation, KIF14, AKT

## Abstract

**Background:**

Ubiquitously transcribed tetratricopeptide repeat, X chromosome (UTX) is an H3K27me3 demethylase, a permissive mark associated with active gene transcription. UTX has been linked to various human cancers. Colorectal cancer (CRC) ranks 3rd among the most common cancers worldwide. However, the role of UTX in colorectal cancer has rarely been reported.

**Methods:**

RT-qPCR, immunoblotting assays (WB), and immunohistochemistry staining were conducted to explore the UTX expression levels in CRC tissues and surrounding normal tissues. CCK-8 assays, colony formation assays, and flow cytometry were also used to determine the potential role of UTX in CRC cell proliferation in vitro. A cell line-derived xenograft model was performed to determine on the role of UTX in HCT116 cell proliferation in vivo. The protein expression levels of UTX, KIF14, AKT, and GAPDH were examined by WB.

**Results:**

Compared with surrounding normal tissues, UTX was upregulated in CRC tissues. Knockdown of UTX significantly inhibited proliferation and caused G0/G1 cell cycle arrest in CRC cell lines, and overexpression of UTX significantly promoted proliferation in CRC cells. Furthermore, knockdown of UTX significantly inhibited tumour growth in vivo. In addition, knockdown of UTX decreased the expression of KIF14 and pAKT and increased the expression of P21.

**Conclusions:**

Our findings indicate that knockdown of UTX inhibits CRC cell proliferation and causes G0/G1 cell cycle arrest through downregulating expression of KIF 14 and pAKT. Thus, UTX may serve as a novel biomarker in CRC.

## Background

Colorectal cancer is the 3rd most common cancer and ranks 4th among the dominant killer cancers worldwide [1, 2]. Epigenetic regulation is widely reported to modulate the aetiology and pathogenesis of CRC [3], and histone methyltransferase has a major role in these processes. For example, as a member of Polycomb repressive complex 2 (PRC2), enhancer of zeste homolog 2 (EZH2) has been found to have a prominent function in colorectal cancer tumourigenesis [4, 5]. However, in many cases, the role of histone demethylase in tumourigenesis still remains unclear, especially with regards to CRC. The interaction between active and inhibitory histone modifications determines gene expression. Generally, the trimethylation of histone H3 lysine 27 (H3K27me3) suppresses gene expression, while the trimethylation of histone H3 lysine 4 (H3K4me3) activates genes expression [6]. In mammalian cells, UTX is one of the primary enzymes that catalyse the demethylation of H3K27me3, a histone marker associated with actively transcribed regions [7], and is generally a tumour suppressor [8]. However, studies have revealed that UTX expression can enhance the proliferation of breast cancer cells [9], although it remains unclear whether and how UTX influences the development of CRC. The primary objective of this study was to determine the role of UTX in tumourigenesis of colorectal cancer and explore its regulatory mechanisms to provide a potential new target for the treatment of colorectal cancer.

KIF14 expression was shown to promote the proliferation of colorectal cancer cells. KIF14 belongs to the kinesin superfamily (KIFs). The proteins are a conserved type of essential molecular motors that are microtubule-dependent and transport transcripts, proteins and organelles while utilizing ATP [10, 11]. At present, at least 45 mammalian KIFs are known and have been classified in 14 separate families [12]. KIF14 is an N-type kinesin of the kinesin-3 superfamily [13, 14] and binds chromatin and microtubules when the bipolar spindle is formed [15–17]. The cell cycle is altered when KIF14 is silenced, thus explaining the role of KIF14 in oncogenesis [17]. The expression level of KIF14 has been reported to be increased in many tumour types, such as glioma, lung cancer, hepatocellular carcinoma, ovarian cancer, breast cancer, and laryngeal carcinoma [17–23], compared to normal tissues. Recently, KIF14 was found to promote cell proliferation via the activation of AKT in colorectal cancer [24].

Here, we reported that UTX expression was elevated in human colorectal cancer and enhanced CRC cell proliferation by promoting the expression of KIF14. Knockdown of UTX inhibited CRC cell proliferation both in vitro and in vivo. Therefore, our studies emphasize that inhibition of UTX activity can be a new strategy for CRC treatment.

## Materials and methods

### Analysis of human specimens

Colorectal cancer specimens were obtained from patients who underwent surgery from 2008 to 2013 in the Shanghai Ninth People's Hospital. None of the patients underwent preoperative chemotherapy or radiation therapy prior to surgery. The study protocol was approved by the ethics committee of the Shanghai Ninth People's Hospital (SH9H-2018-A662-1). In the follow-up of 206 cases, 155 cases returned and 51 cases were lost to follow-up. The actual follow-up rate was 75.24%. From the specimen library, 30 patients with paracancerous tissues were used to assess UTX expression in tumour tissues compared with the normal counterparts. Eighty-seven patients with staging formation were chosen to analyse UTX expression in different stages of colorectal cancer. Dukes staging was used in our study. Tissue microarrays used in this study included 105 CRC specimens with survival information grouped according to the UTX expression level. The scoring method of the tissue microarray has been described in “Materials and methods” section in red. Fundamentally, the quantification method was a multiplicative index of staining proportion and staining extent in the cores confirmed by two independent pathologists. Scores ≥ 2 were considered to be high levels of UTX, while scores < 2 were considered to be low levels of UTX.

### Immunohistochemical (IHC) analysis

For colorectal cancer patient samples and mouse tumour-forming tissue samples, we first performed paraffin embedding. Immunohistochemical staining was performed on 4-μm sections of paraffin-embedded tissues to determine the expression level of UTX protein. In brief, the slides were incubated in UTX (33510, CST) and KIF14 antibody (ab71155, Abcam) and diluted 1:200 at 4 °C overnight. Haematoxylin staining was performed to identify nuclei. Images were acquired using a Leica DM 4000B microscope.

### Extraction of RNA and q-PCR

We extracted total RNA using RNAiso Plus reagent (TaKaRa), and 1.0 µg RNA was subjected to reverse transcription using a RT-PCR kit (Perfect Real Time, TaKaRa) as instructed by the manufacturer. An ABI-7500 Fast Real-Time PCR Detection System was used to perform all qRT-PCR experiments. GAPDH was used as a control to normalize the amplified transcript level of each indicated gene. The primers used were as follows:

UTX: F: GAACAGCTCCGCGCAAATAG
  R: CGTACCTGTGCAACTCCTGT
GAPDH: F: TCTGATTTGGTCGTATTGGG
  R: GGAAGATGGTGATGGGATT

### CCK-8 assay

We performed the CCK-8 assay using a CCK-8 kit (Dojindo, Japan) as instructed by its manufacturer. In short, we seeded a total of 2000 cells into each well in a 96-well plate. We then added 10 µl CCK-8 solution to each well and incubated the cells for 2–4 h in the incubator. We measured the absorbance at 450 nm of the plate using a microplate reader.

### Colony formation assay

We seeded 1000 cells into one well of 6-well plates. Crystal Violet Staining Solution (Beyotime C0121) was used to stain the cells after 10 days. Then, we counted colonies containing ≥ 20 cells.

### Flow cytometry

We seeded 1 × 10^5^ stable UTX knockdown and control cells into a six-well plate, and the cells were later harvested after 48 h and subjected to flow cytometry. Cell cycle analysis was performed with the Cell Light Edu Kit (C10338: Ruibo) as instructed by the manufacturer.

### Tumourigenesis assay

We injected 0.2 ml from medium of lentivirally transduced HCT116 cells (1–3 × 10^6^) subcutaneously into the right armpits of the nude mice. We then evaluated the size of the tumour after 3 days and used the formula V = 1/2(a * b * b) to calculate tumour volume, where *a* and *b* denote the major and minor tumour axis, respectively. The tumour weight was quantified 3 to 4 weeks after injection when the mice were killed. We strictly followed the European Community guidelines for the use of experimental animals (86/609/EEC) when handling the experimental animals.

### Immunoblotting assay

Cells were lysed using a loading lysis buffer. Lowry protein assays were used to calculate protein concentration. Then, we separated 30 μg of protein lysate using SDS-PAGE analysis. The protein was transferred to a PVDF membrane (Immobilon P; Millipore, Milford, MA). The indicated protein was detected by immunoblotting with specific antibodies in 5% albumin from bovine serum. The antibody used were as follows: UTX (33510, CST), KIF14 (ab71155, Abcam), pAKT S473 (4501, CST), pAKT T308 (4506, CST), AKT (4658, CST), and P21 (2947, CST).

### Statistical analysis

All of the results were repeated at least thrice except when indicated otherwise. The mean ± SEM of repeats was used to represent the data. To compare the differences between groups, 2-tailed paired Student’s t-test was applied. We then used Pearson’s correlation test to determine the correlation between UTX and KIF14 in human tissues. Log-rank tests were used to calculate the Kaplan–Meier survival curve comparison. We did not exclude any material or data in the study when doing the statistical analysis.

## Results

### UTX expression is upregulated in human CRC

**Fig. 1:**
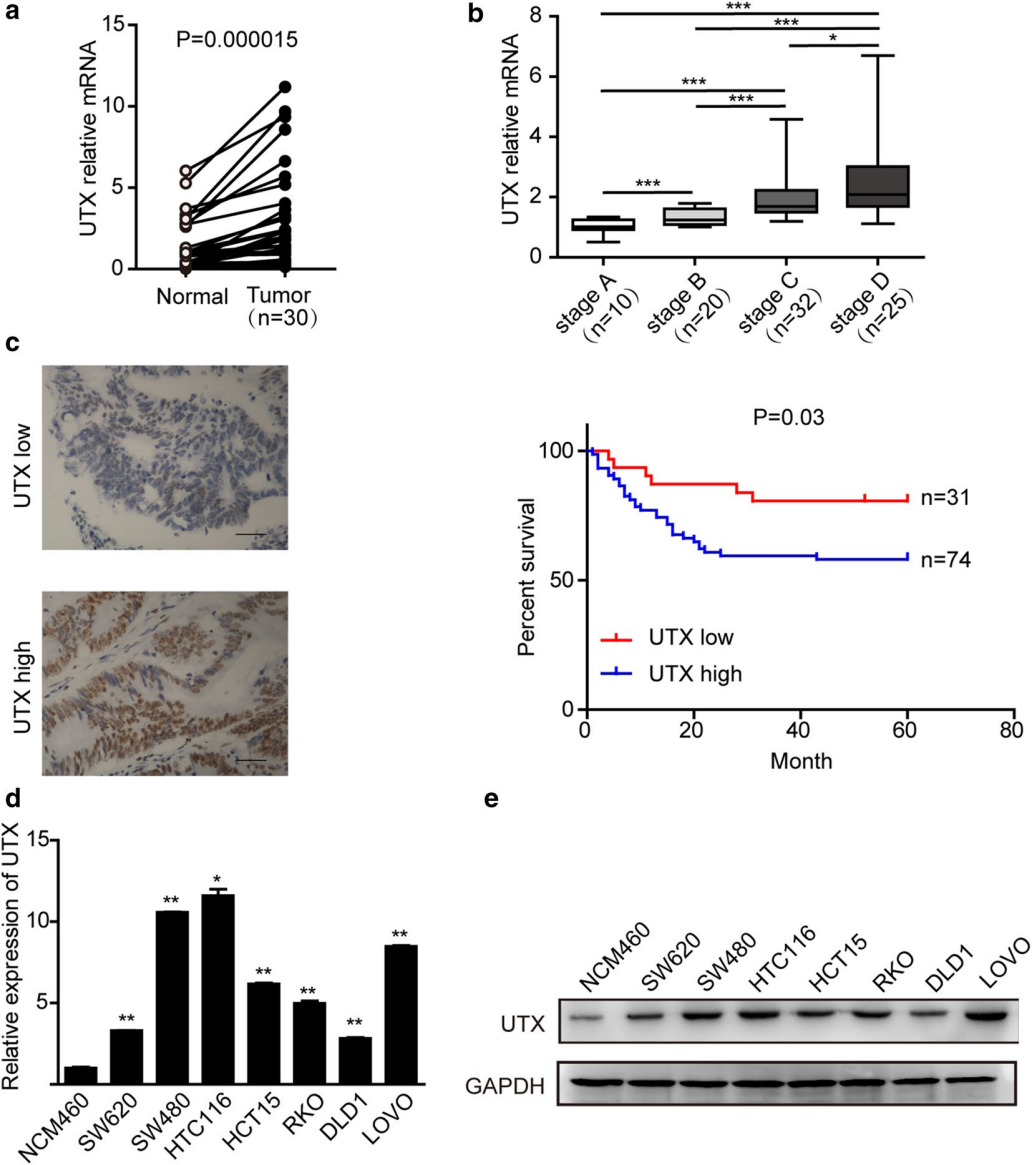
Relevance of UTX expression in human CRC. **a** UTX mRNA levels in 30 matched tumour and paracarcinoma tissues (paired t test). **b** Boxed plot of UTX mRNA expression assessed by real-time PCR at different clinical stages. **c** Kaplan–Meier plot of overall survival of patients based on UTX levels. A log-rank test was used for statistical analysis. **d** UTX mRNA levels in different CRC cell lines. **e** UTX protein levels in different CRC cell lines. All the experiments were performed at least three times. Data are shown as the mean ± SEM. (*p < 0.05, **p < 0.01, ***p < 0.001, Student’s t-test)

To determine the important role played by UTX in CRC, we first examined the RNA level of UTX in CRC patients using quantitative reverse-transcriptase PCR (RT-qPCR). From the results, UTX was significantly upregulated in CRC tissues compared to the surrounding normal tissues (Fig. 1a). Correlation studies also indicated that the expression level of UTX in patients with CRC was proportional to the stage of disease progression (Fig. 1b). Simultaneously, we used immunohistochemical detection of a colorectal cancer tissue microarray to detect the expression of UTX. Based on the expression level of UTX, we separated all the samples into two groups. We found that the group with a higher expression of UTX had lower survival rates than the other group (Fig. 1c). We also detected UTX expression in normal colorectal NCM460 cells and several colorectal cancer cell lines. We found that the RNA and protein levels of UTX in colorectal cancer cell lines were significantly higher than those of the normal colorectal NCM460 cells (Fig. 1d, e). These data suggested that UTX can be a potential biomarker for colon cancer and indicated that there is a causal relationship between UTX and tumourigenesis of CRC.

### UTX promotes CRC cell proliferation

**Fig. 2:**
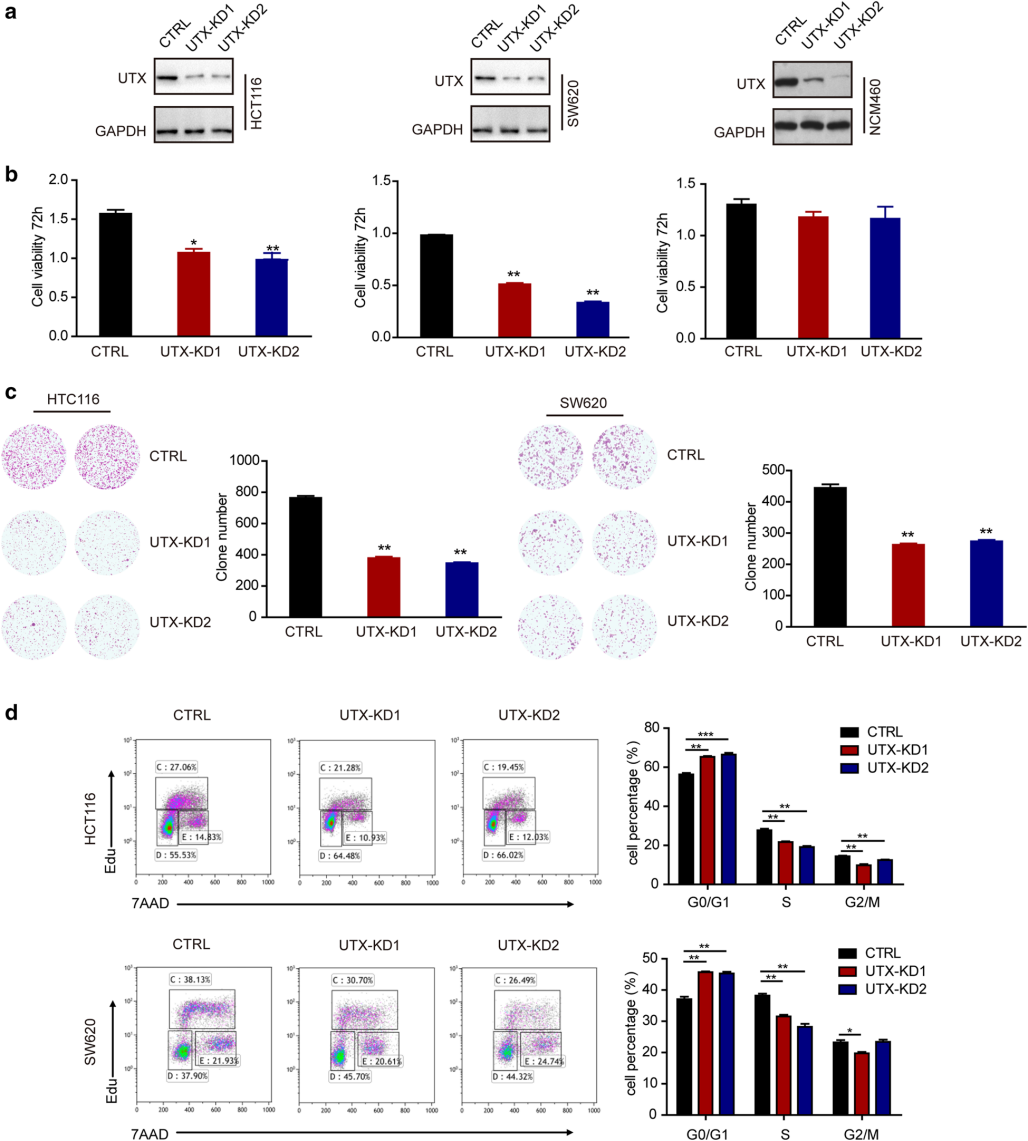
Knockdown of UTX inhibits cell proliferation in HCT116 and SW620 cell lines. **a** Western blotting analysis of the indicated proteins in control (scrambled) and UTX knockdown (UTX-KD) HCT116 cells or SW620 cells. **b** CCK8 assays of CTRL and UTX-KD HCT116 cells or SW620 cells were performed. **c** Colony formation assays of CTRL and UTX-KD HCT116 cells or SW620 cells were performed. **d** The effects of UTX on cell-cycle arrest were evaluated by flow cytometry in CTRL and UTX-KD HCT116 cells or SW620 cells. All the experiments were performed at least three times. Data are shown as the mean ± SEM. (*p < 0.05, **p < 0.01, ***p < 0.001, Student’s t-test)

**Fig. 3:**
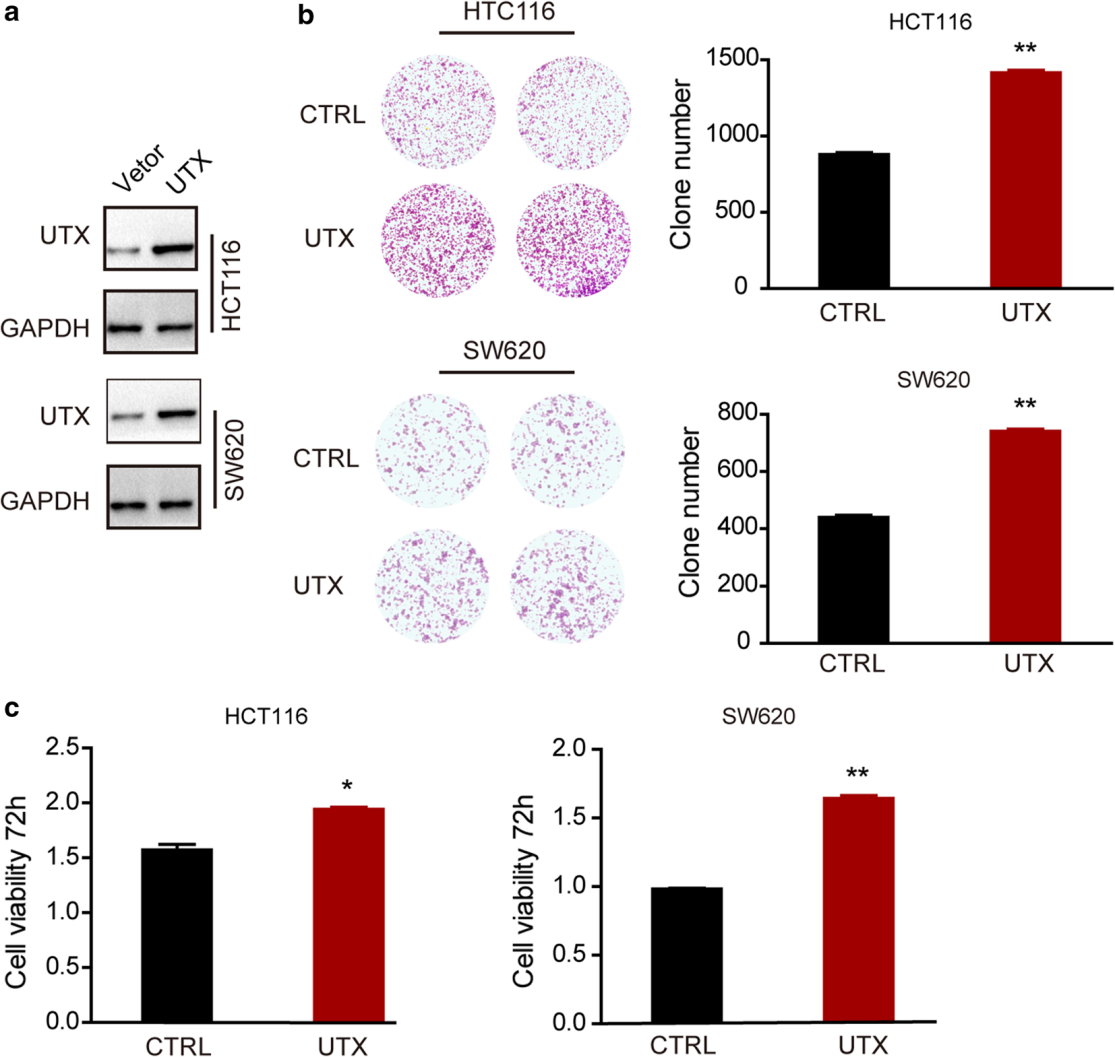
Overexpression of UTX promotes cell proliferation in HCT116 and SW620 cell lines. **a** Western blotting analysis of the indicated proteins in control (scrambled) and UTX overexpression (UTX) HCT116 cells or SW620 cells. **b** Colony formation assays of CTRL and UTX HCT116 cells or SW620 cells were performed. **c** CCK8 assays of CTRL and UTX HCT116 cells or SW620 cells were performed. All experiments were performed at least three times. Data are shown as the mean ± SEM. (*p < 0.05, **p < 0.01, ***p < 0.001, Student’s t-test)

To examine the effect of UTX in CRC cell lines, we generated UTX knockdown HCT116, SW620 and normal epithelial NCM460 cells by two shRNAs. The expression of UTX decreased after infection with shRNA lentivirus in both cell lines (Fig. 2a). We found that UTX depletion inhibited the growth of CRC cells using CCK-8 and colony formation assays (Fig. 2b, c). However, UTX depletion had no effect on proliferation of colonic epithelial NCM460 cells (Fig. 2b, c). These results indicated that UTX promoted CRC progression in colon cancer cell lines. We then examined the cell cycle distribution of colorectal cancer cell lines after UTX knockdown. As expected, the downregulation of UTX suppressed the cell cycle transition at G1/S phase in both cell lines (Fig. 2d). These data indicate that UTX promotes colorectal cancer cell proliferation by affecting the distribution of the cell cycle. We also performed UTX overexpression in two colorectal cancer cell lines, HCT116 and SW620 (Fig. 3a). By CCK-8 and colony formation assays, we found that increased UTX expression promoted the proliferation of CRC cells (Fig. 3b, c).

### UTX depletion inhibits xenograft growth in vivo

**Fig. 4:**
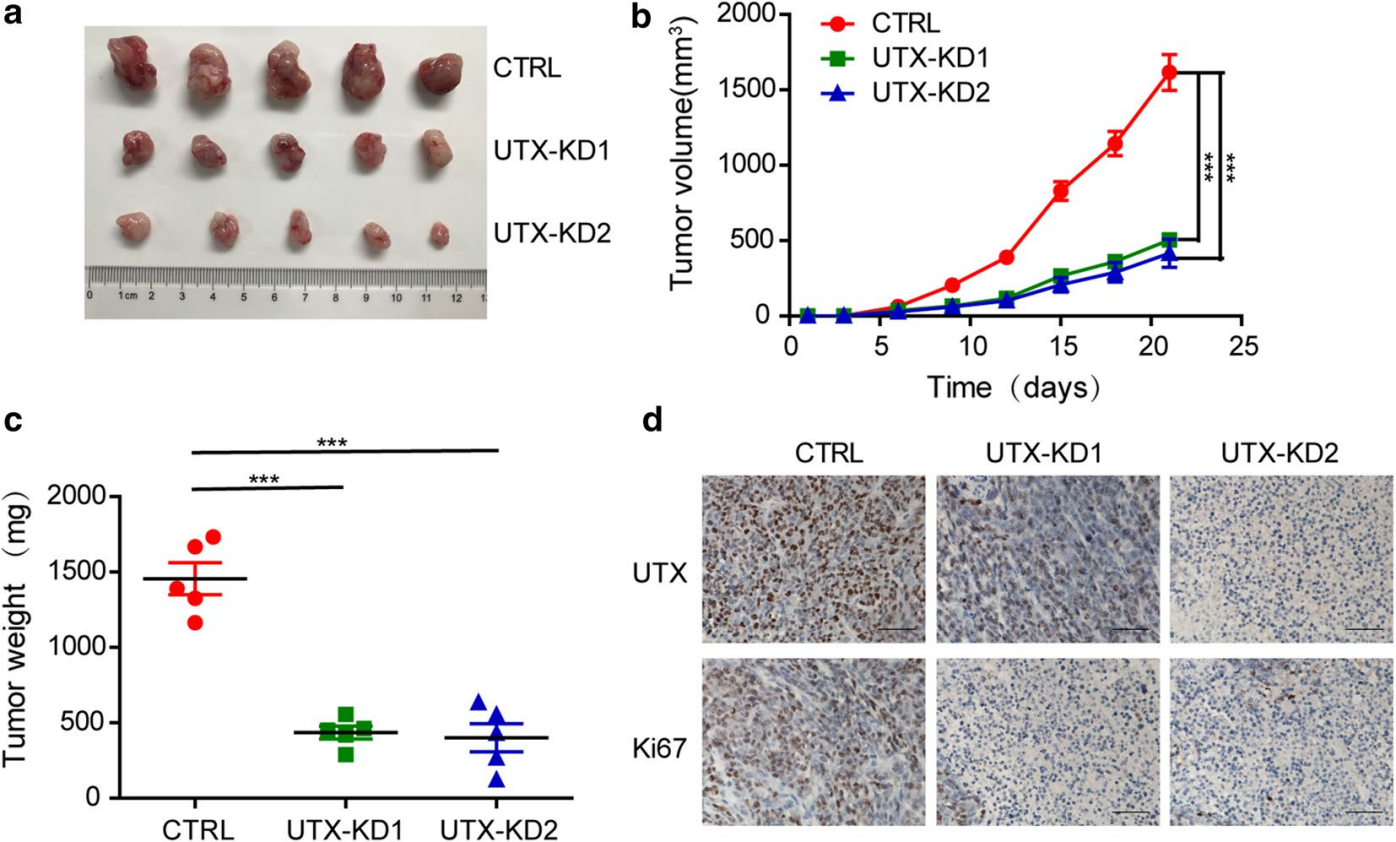
UTX is essential for CRC growth in vivo. A total of 2 * 10^6^ cells were subcutaneously injected into the left dorsal flank per nude mouse. **a** UTX knockdown suppressed xenograft tumour growth of HCT116 cells in vivo (n = 5). Images of tumours are from all mice in each group. **b** Tumour volume was measured. **c** Tumour weight was measured. **d** Representative photographs of UTX and Ki-67 IHC staining in the indicated tumours. Data are shown as the mean ± SD. (*p < 0.05, **p < 0.01, ***p < 0.001, Student’s t-test)

Then, we used xenograft mouse models to determine the effect of UTX on colorectal cancer tumourigenesis. HCT116 cells were transfected with the pLVX-shRNA1 plasmid with UTX shRNA or a scrambled sequence. We then screened the stable cell lines. These cells were administered to the nude mice via subcutaneous injection. After the mice were sacrificed 3 to 4 weeks later, the volume and weight of the tumours were measured. In addition, we found that knockdown of UTX was associated with a reduction in both tumour volume and weight (Fig. 4a–c). In addition, there were fewer Ki67-positive cells in UTX knockdown tumours compared to the control group (Fig. 4d). From the results, it can be deduced that UTX promotes tumour development by promoting cell proliferation in vivo.

### UTX promotes cell proliferation via activation of AKT required expression of KIF14 in colorectal cancer

**Fig. 5:**
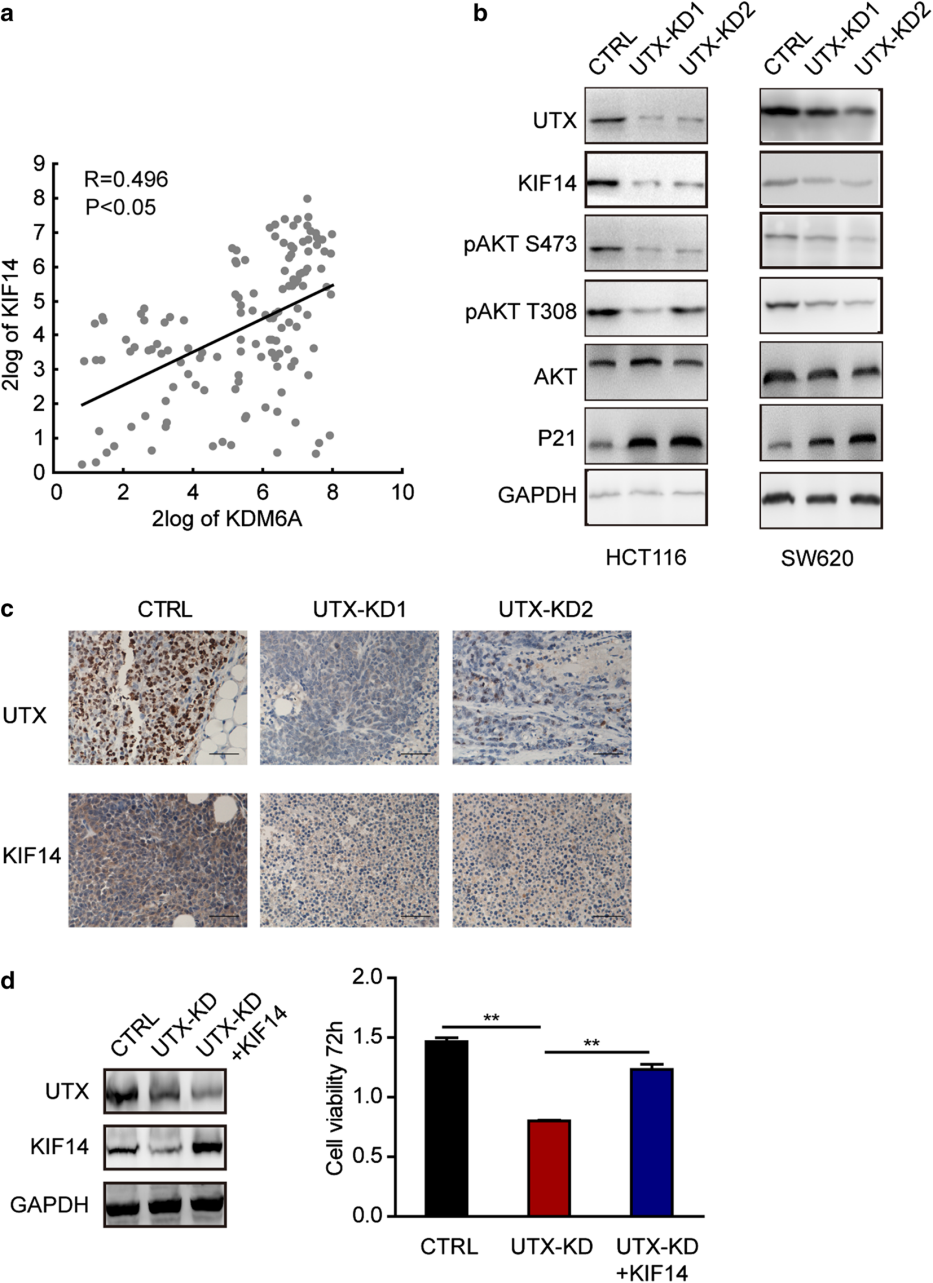
UTX regulates ATK activation by regulating KIF14 in HCT116 and SW620 cells in vitro. **a** Correlation between UTX and KIF14 levels in CRC patients (n = 135). Each point represents one independent sample, and the correlation coefficient (R) and p value are presented. **b** Western blotting analysis of UTX, KIF14, pAKT (S473, T308) and other indicated proteins in HCT116 and SW620 cells. **c** Representative photographs of UTX and KIF14 IHC staining in the indicated tumours. All the experiments were performed at least three times. **d** CCK8 assays was used to detected proliferation of HCT116 cells after transfection with shCtrl, shUTX lentivirus and shUTX lentivirus plus KIF14 overexpression lentivirus

Upregulated KIF14 expression has been reported in a variety of tumours, [17–23]. More recently, KIF14 was found to promote cell proliferation via the activation of AKT in colorectal cancer [24]. Based on the fact that KIF14 is a latent driver of growth in CRC tumourigenesis, we suggested that UTX might upregulate KIF14 and activate AKT signalling, thus promoting the progression of CRC. To assess the correlation between UTX and KIF14, we measured the RNA expression of UTX and KIF14 simultaneously in 135 CRC tissues using RT-qPCR. We found a dramatic (p < 0.05) positive correlation between the expression of UTX and KIF14 (Fig. 5a). We also confirmed the protein expression levels of KIF14 and its downstream proteins pAKT (phosphorylated AKT, S473 and T308) and P21 in UTX-KD and control CRC cell lines using WB (Fig. 5b). We found that pAKT decreased in UTX-KD cells compared to control cells. To further confirm that KIF14 was regulated by UTX in CRC, we examined the expression of UTX and KIF14 in xenograft tumours using immunohistochemistry (IHC) staining. Lower KIF14 expression was observed in UTX-KD tumours (Fig. 5c). We then overexpressed KIF14 after knocking down UTX, and we found that overexpression of KIF14 could significantly rescue the cell proliferation defects caused by UTX knockdown (Fig. 5d). These results indicated that UTX promotes cell proliferation through expression of KIF14 in colorectal cancer.

## Discussion

KIF14 is upregulated in multiple types of tumours and act as an oncogene. Sustained high expression promotes tumour cell proliferation [17–23]. Therefore, the specific targeting of KIF14 to inhibit tumour cell proliferation can be used to yield therapeutic benefits. Here, we showed that UTX is highly expressed in CRC cells, and the expression level of UTX was positively correlated with the degree of progression of CRC. UTX expression served as an independent predictor for risk stratification of overall survival in CRC patients. Knockdown of UTX inhibited CRC cell proliferation and caused cell cycle arrest at the G0/G1 phase by downregulating the expression of KIF14 and pAKT. Therefore, UTX can be a new diagnostic biomarker and a potential therapeutic target in the management of CRC.

So far, there have been few reports on the role of UTX in cancer [8]. Most studies suggest that UTX is a tumour suppressor gene, and there are reports that UTX can promote tumourigenesis [9]. The function of UTX in colorectal cancer has not yet been elucidated. Only one study showed that UTX can induce E-cad and promote colorectal cancer metastasis [25]. Nevertheless, we observed that the morphology of CRC cell lines after UTX knockdown did not change significantly, but the cell growth was significantly slower in our study. The mechanism of UTX regulation of colorectal cancer cell proliferation in this study also has some shortcomings. Although we found that the expression of KIF14 was regulated by UTX, ChIP experiments showed that there was no obvious H3K27me3 enrichment in the promoter region of KIF14, indicating that KIF14 is indirectly regulated by UTX. Thus, identification of a key gene, which is directly regulated by UTX, that regulates KIF14 expression, further exploration is still needed.

Furthermore, UTY, a homologous gene of UTX, has been considered to have no demethylase activity, but recent studies have shown that UTY can partially compensate for UTX demethylase activity in UTX knockout mice [26]. Therefore, considering the difference in incidence of colorectal cancer between different sexes, it is important to study the role of UTY in colorectal cancer. Whether UTY also promotes the development of colorectal cancer by participating in the regulation of KIF14 expression requires further research. At the same time, the role of JMJD3, which is the same as UTX in the demethylation of H3K27, in colorectal cancer is still unclear. The mutual compensatory effects of the two enzymes in the pathogenesis of colorectal cancer also require further study.

In summary, we first reported the high expression of UTX in colorectal cancer and found its regulation of KIF14 expression. The relevance of this regulation in the aetiology and the pathogenesis of CRC yields a new view on how epigenetics and tumourgenesis are related. Inhibition of colorectal cancer cell proliferation by inhibiting UTX enzyme activity may be a potential new therapeutic strategy that can be used for the benefit of patients with CRC.

## Conclusions

In summary, we demonstrated that UTX is highly expressed in CRC cells, and the expression of UTX is positively correlated with the degree of progression of CRC. Patients with CRC that express high levels of UTX have shorter survival. Knockdown of UTX inhibited CRC cell proliferation and caused cell cycle arrest at G0/G1 phase by downregulating the expression of KIF14 and pAKT. Therefore, UTX can serve as both a new diagnostic biomarker and a potential target for CRC therapy.

## Abbreviations

UTX: ubiquitously transcribed tetratricopeptide repeat, X chromosome
H3K27me3: trimethylation of histone H3 lysine 27
H3K4me3: trimethylation of histone H3 lysine 4
RT-qPCR: reverse transcription quantitative polymerase chain reaction
IHC: immunohistochemistry
WB: immunoblotting assays
CRC: colorectal cancer
FBS: foetal bovine serum
ChIP: chromatin immunoprecipitation
PRC2: Polycomb repressive complex 2
EZH2: enhancer of zeste homolog 2
PBS: phosphate-buffered saline
KIFs: kinesin superfamily
KDM: lysine-specific demethylase
